# Diverse Strategies for Modulating Insulin Resistance: Causal or Consequential Inference on Metabolic Parameters in Treatment-Naïve Subjects with Type 2 Diabetes

**DOI:** 10.3390/medicina60060991

**Published:** 2024-06-17

**Authors:** Eiji Kutoh, Alexandra N. Kuto, Rumiko Okada, Midori Akiyama, Rumi Kurihara

**Affiliations:** 1 Biomedical Center, Tokyo 132-0034, Japan; 2 Division of Diabetes and Endocrinology, Department of Internal Medicine, Gyoda General Hospital, Saitama 361-0056, Japan; 3 Division of Diabetes and METABOLISM, Department of Internal Medicine, Higashitotsuka Memorial Hospital, Yokohama 244-0801, Japan; 4 Division of Diabetes, Department of Internal Medicine, Kumagaya Surgical Hospital, Kumagaya 360-0023, Japan

**Keywords:** insulin resistance, very low-calorie Japanese diet, pioglitazone, SGLT-2 inhibitor

## Abstract

*Bacground and Objectives*: The objective of this study is to investigate how different therapies modulating insulin resistance, either causally or consequently, affect metabolic parameters in treatment-naïve subjects with T2DM. *Subjects and Methods*: A total of 212 subjects were assigned to receive either a tight Japanese diet (*n* = 65), pioglitazone at doses ranging from 15–30 mg/day (*n* = 70), or canagliflozin at doses ranging from 50–100 mg/day (*n* = 77) for a duration of three months. Correlations and changes (Δ) in metabolic parameters relative to insulin resistance were investigated. *Results*: Across these distinct therapeutic interventions, ΔHOMA-R exhibited significant correlations with ΔFBG and ΔHOMA-B, while demonstrating a negative correlation with baseline HOMA-R. However, other parameters such as ΔHbA1c, ΔBMI, ΔTC, ΔTG, Δnon-HDL-C, or ΔUA displayed varying patterns depending on the treatment regimens. Participants were stratified into two groups based on the median value of ΔHOMA-R: the lower half (X) and upper half (Y). Group X consistently demonstrated more pronounced reductions in FBG compared to Group Y across all treatments, while other parameters including HbA1c, HOMA-B, TC, TG, HDL-C, non-HDL-C, TG/HDL-C ratio, or UA exhibited distinct regulatory responses depending on the treatment administered. *Conclusions*: These findings suggest that (1) regression to the mean is observed in the changes in insulin resistance across these therapies and (2) the modulation of insulin resistance with these therapies, either causally or consequentially, results in differential effects on glycemic parameters, beta-cell function, specific lipids, body weight, or UA.

## 1. Introduction

Insulin resistance and beta-cell function constitute pivotal components in the pathophysiology of Type 2 diabetes (T2DM). Insulin resistance denotes a state wherein cellular responsiveness to insulin diminishes, culminating in elevated blood glucose levels [1,2]. While beta-cell function involves a singular organ (the pancreas) and hormone (insulin), insulin resistance presents a complex scenario, entailing the participation of diverse molecules and signal transduction pathways in various organs such as adipose tissue, liver, or kidney [1,2,3]. Moreover, insulin resistance may inflict damage upon beta-cells, thereby impairing beta-cell function [3].

The mitigation of insulin resistance in obese individuals with T2DM primarily involves body weight control through dietary interventions and/or exercise. Additionally, certain drugs, such as thiazolidinedione (TZD) and SGLT-2 inhibitors, have demonstrated favorable impacts on insulin resistance [4,5].

Pioglitazone, classified as a TZD oral hypoglycemic agent, operates by activating peroxisome proliferator-activated receptor gamma (PPAR-γ), thereby regulating the expression of factors that contribute to insulin sensitivity in adipose tissue, liver, and muscle [4]. Notably, pioglitazone has exhibited the capacity to enhance beta-cell function and elicit favorable effects on lipid profiles [4,6]. However, its use has diminished due to associated adverse events, including weight gain and a suspected increase in the incidence of bladder cancer in men [4]. Despite these concerns, pioglitazone is currently under re-evaluation owing to its beneficial cardiovascular effects [7].

Canagliflozin, an SGLT-2 inhibitor, functions by impeding glucose reabsorption in the kidneys, thereby augmenting urinary glucose excretion [5]. As anticipated from their mechanism of action, SGLT-2 inhibitors induce weight loss [5]. Furthermore, SGLT-2 inhibitors are recognized for their favorable effects on insulin resistance, beta-cell function, and specific lipid profiles [5,8]. Intriguingly, it has been demonstrated that the weight loss induced by one SGLT-2 inhibitor, canagliflozin, is not inherently associated with insulin-sensitizing properties or glycemic efficacy [8].

Currently, the association between changes in insulin resistance using these methods and alterations in other diabetic parameters remains unclear. In this context, the implementation of a very low-calorie (tight) Japanese diet, pioglitazone, and canagliflozin emerges as an intriguing investigative strategy. All three approaches are acknowledged to reduce insulin resistance and glycemic parameters, yet they manifest distinct effects on other parameters such as beta-cell function, weight, and lipid profiles. While the hyperinsulinemic-euglycemic clamp and intravenous glucose tolerance test represent the most reliable methods for estimating insulin resistance, their feasibility within routine clinical settings is constrained [9]. Consequently, the HOMA-R index, a mathematical model strongly correlating with the hyperinsulinemic-euglycemic clamp procedure, has been employed to assess systemic insulin resistance across numerous studies [10]. In this study, we have selectively examined various diabetic parameters closely associated with T2DM, investigating their correlations and regulatory patterns relative to insulin resistance through the employment of three distinct therapeutic strategies.

## 2. Subjects and Methods

### 2.1. Subjects

The subjects were recruited from the outpatient divisions of the affiliated hospitals of the first author (EK). Primarily sourced from the annual health check screening system, inclusion criteria mandated that participants were either newly diagnosed with T2DM or previously diagnosed but untreated. The subjects had not received any regularly prescribed medications in the six months preceding the study. Exclusion criteria encompassed clinically significant renal impairment (creatinine > 1.5 mg/dL), hepatic dysfunction (glutamic oxalacetic transaminases/glutamic pyruvic transaminases [AST/ALT] > 70/70 IU/L), a history of heart disorders, severe hypertension (systolic blood pressure > 160 mm Hg and/or diastolic blood pressure > 100 mm Hg), Type 1 Diabetes Mellitus (T1DM), and pregnancy. The specifics of the very low-calorie/carbohydrate Japanese diet were previously elucidated by Japanese researchers [11,12,13]. Briefly, (1) calories do not exceeding 25 kcal/kg/day, (2) prioritize fish consumption over meat, and (3) prioritize vegetables or protein at the beginning of the meal, followed by carbohydrates such as rice, noodles, or bread. Male participants were administered a tight Japanese diet (*n* = 40), 30 mg/day pioglitazone (*n* = 53), or 100 mg/day canagliflozin (*n* = 59) as monotherapy. Female participants received a tight Japanese diet (*n* = 15), 15 mg/day pioglitazone (*n* = 17), or 50 mg/day canagliflozin (*n* = 18), owing to adverse events being more prevalent in women (e.g., edema with pioglitazone, urogenital infections with SGLT-2 inhibitors). Adherence to the study protocol was monitored during clinic visits. Participants who dropped out were excluded from the data analysis. The assignment was not strictly randomized; hence, this project entails the comparison of three observational studies. Informed consent was obtained from the participants, and the study protocol received approval from the Ethical Committee/Institutional Review Board of Gyoda General Hospital and Kumagaya Surgery Hospital. This study adhered to the principles of the Helsinki Declaration and Good Clinical Practice.

### 2.2. Laboratory Measurements

The primary endpoint pertained to the changes in HOMA-R from baseline to 3 months. Secondary endpoints encompassed changes in FBG, HbA1c, insulin, HOMA-B, T-C, TG, HDL-C, TG/HDL-C, non-HDL-C, UA, and BMI over the same period. Fasting blood samples were collected in the morning. Monthly measurements of HbA1c and FBG were performed, while insulin, T-C, TG, HDL-C, and UA were measured at both the study’s commencement (baseline) and conclusion (3 months). In some patients, antiglutamic acid decarboxylase (GAD) antibodies were assayed to exclude those with T1DM (Mitsubishi LSI or BML, Tokyo, Japan). HOMA-R and HOMA-B were calculated as previously described [10]: HOMA-R = insulin × FBG/405, HOMA-B = insulin × 360/(FBG-63).

### 2.3. Data Analyses

Statistical analysis was conducted using the PAST program developed by the University of Oslo (https://folk.uio.no/ohammer/past/ accessed through 3 January 2024 to 28 February 2024). Unpaired Student’s *t*-tests were employed to assess baseline value differences, while paired Student’s *t*-tests were utilized to analyze intra-group differences. Simple regression analysis was performed to investigate correlations between baseline or changes in HOMA-R and diabetic parameters. Analysis of covariance (ANCOVA) was employed to determine inter-group differences in diabetic parameter changes. Throughout the statistical analysis, significance was assigned to values of *p* < 0.05, and values within the range of 0.05 < *p* < 0.1 were considered statistically insignificant but suggestive of potential differences or correlations, as per established methodology [14].

## 3. Results

### 3.1. Baseline Characteristics and Associations between Insulin Resistance and Diabetic Parameters in Newly Diagnosed, Treatment-Naïve Subjects with Type 2 Diabetes at Baseline (All Subjects)

The baseline characteristics of all the enrolled subjects are shown in Table 1.

Significant correlations were discerned between HOMA-R and various parameters at baseline, including FBG (R = 0.295), HOMA-B (R = 0.535), BMI (R = 0.466), insulin (R = 0.886), and UA (R = 0.279), whereas negative correlations manifested between HOMA-R and age (R = −0.145). TG (R = 0.127, *p* = 0.064). TG/HDL-C (R = 0.120, *p* = 0.081) exhibited a tendency towards positive correlations, while HDL-C (R = −0.131, *p* = 0.056) displayed a tendency towards a negative correlation with HOMA-R (Table 2).

Subsequently, subjects were stratified into two groups based on the median baseline values of HOMA-R, yielding lower half (group A) and upper half (group B) designations. As depicted in Table 3, group B exhibited significantly elevated levels of HOMA-R, FBG, insulin, HOMA-B, BMI, and UA and concurrently lower levels of age and HDL-C in comparison to group A. TG, TG/HDL-C, and non-HDL-C displayed a propensity to be higher in group B relative to group A (*p* = 0.099 and *p* = 0.051, respectively). Conversely, HbA1c demonstrated no discernible differences between these two groups, if any.

### 3.2. Alterations in Diabetic Parameters following Very Low-Calorie (Tight) Japanese Diet, Pioglitazone, or Canagliflozin Monotherapy in Treatment-Naïve Subjects with T2DM

At baseline, no significant differences in these diabetic parameters were observed among the three treatment groups (data not presented as a table).

After 3 months, significant reductions in FBG, HbA1c, and HOMA-R, along with increases in HOMA-B, were evident across all three treatment groups. Conversely, diverse regulatory patterns were observed in other parameters. Under the tight Japanese diet regimen, T-C, non-HDL-C, and BMI exhibited significant decreases, while UA increased (Table 4A; for each value and statistical significance, refer to the corresponding tables). Pioglitazone resulted in significant reductions in TG and TG/HDL, coupled with increases in HDL-C and BMI (Table 4B). Canagliflozin yielded a significant increase in HDL-C, accompanied by a decrease in BMI. TG exhibited a tendency to decrease (Table 4C).

### 3.3. Correlation between Changes in Insulin Resistance and Diabetic Parameters with Very Low Calorie (Tight) Japanese Diet, Pioglitazone or Canagliflozin

Simple regression analysis was conducted to examine the relationships between alterations in insulin resistance (ΔHOMA-R) and corresponding changes in other diabetic parameters under the three treatment strategies.

**Figure 1:**
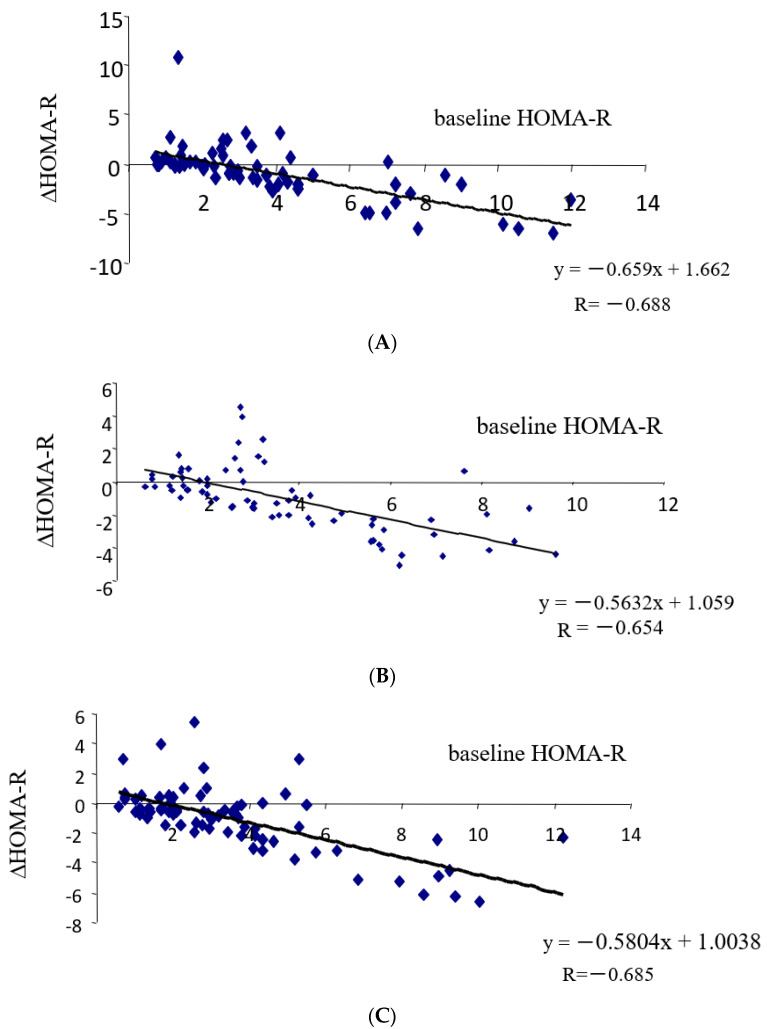
Baseline-dependent regulation of insulin resistance Simple regression analysis was performed between the changes in (Δ) HOMA-R and baseline HOMA-R. (**A**) Tight Japanese diet; (**B**) pioglitazone; (**C**) canagliflozin.

With tight Japanese diet, as delineated in Table 5A, significant correlations were observed between ΔHOMA-R and changes in ΔFBG (R = 0.599), ΔHbA1c (R = 0.256), Δinsulin (R = 0.932), or ΔHOMA-B (R = 0.452). Marked negative correlations were noted between ΔHOMA-R and baseline HOMA-R (R = −0.688, Figure 1A). Insignificant negative correlations were observed between ΔHOMA-R and ΔUA (R = −0.217, *p* = 0.082).

With pioglitazone, as illustrated in Table 5B, significant correlations were identified between ΔHOMA-R and changes (Δ) in FBG (R = 0.510), ΔHbA1c (R = 0.266), Δinsulin (R = 0.771), ΔHOMA-B (R = 0.298), ΔT-C (R = 0.283), ΔTG (R = 0.299), and Δnon-HDL-C (R = 0.260). Significant negative correlations were observed between ΔHOMA-R and baseline HOMA-R (R = −0.654, Figure 1B) and ΔBMI (R = −0.342). A tendency of correlations was observed between ΔHOMA-R and ΔTG/HDL-C (R = 0.230, *p* = 0.055).

With canagliflozin, as depicted in Table 5C, significant correlations were observed between ΔHOMA-R and ΔFBG (R = 0.322), Δinsulin (R = 0.849), ΔHOMA-B (R = 0.365), and ΔTG/HDL-C (R = 0.293). No correlations, if any, were observed between ΔHOMA-R and ΔHbA1c. Significant negative correlations were seen between ΔHOMA-R and baseline HOMA-R (R = −0.685, Figure 1C). Insignificant positive or negative correlations were observed between ΔHOMA-R and ΔTG (R = 0.215, *p* = 0.072) and ΔUA (R = −0.196, *p* = 0.093), respectively.

### 3.4. Differential Regulations of Diabetic Parameters in Two Groups with Distinct Changes in Insulin Resistance

Within each treatment group, subjects were stratified into two subgroups based on the median value of the changes (Δ) in HOMA-R: lower ΔHOMA-R (group X) and higher ΔHOMA-R (group Y), as detailed in the Subjects and Methods section. Notably, in each treatment group, baseline HOMA-R was significantly higher in group X compared to group Y (Table 6(AX,AY,BX,BY,CX,CY)).

**Figure 2:**
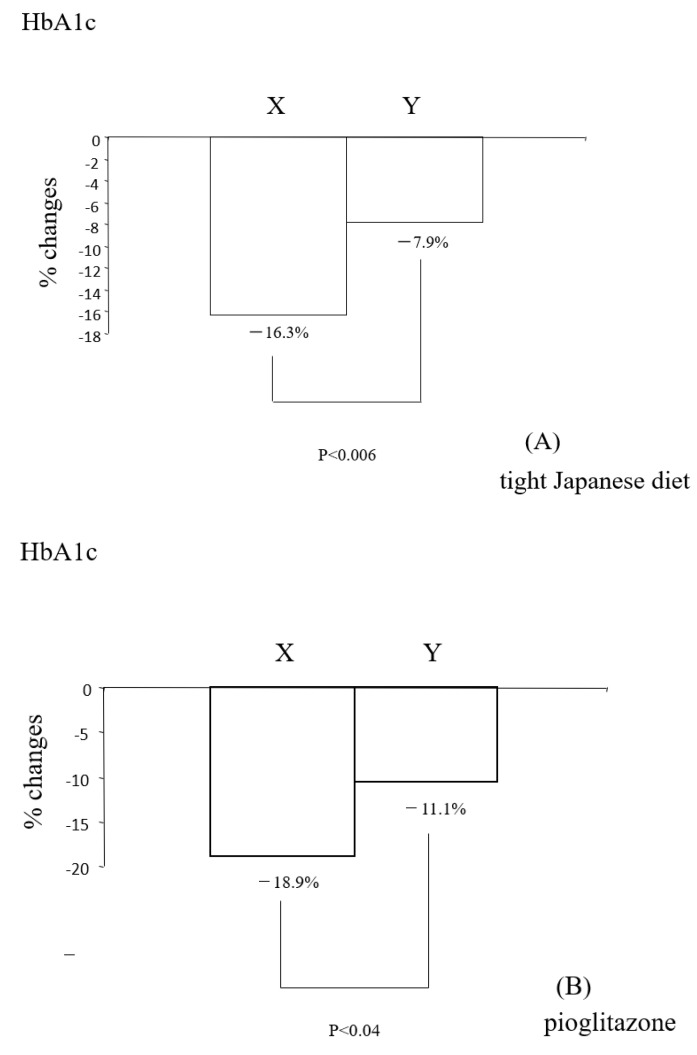

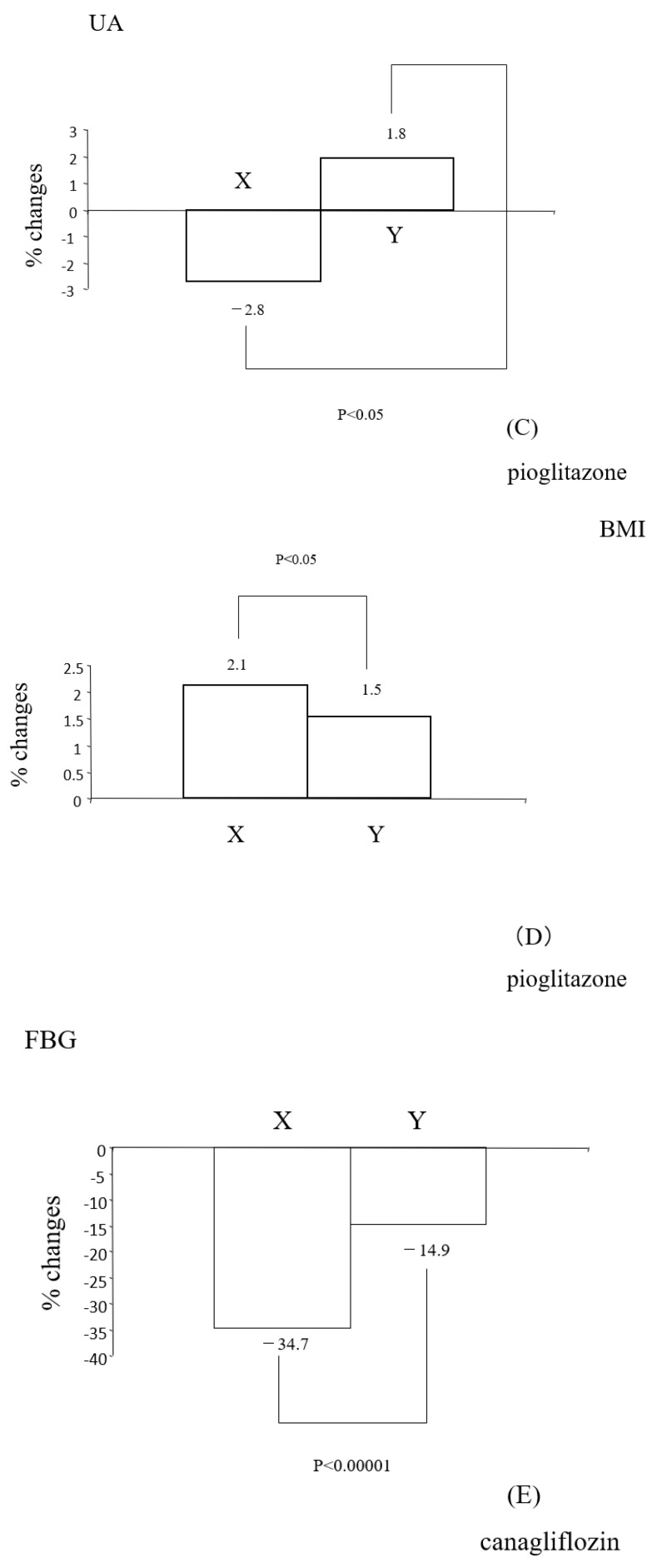

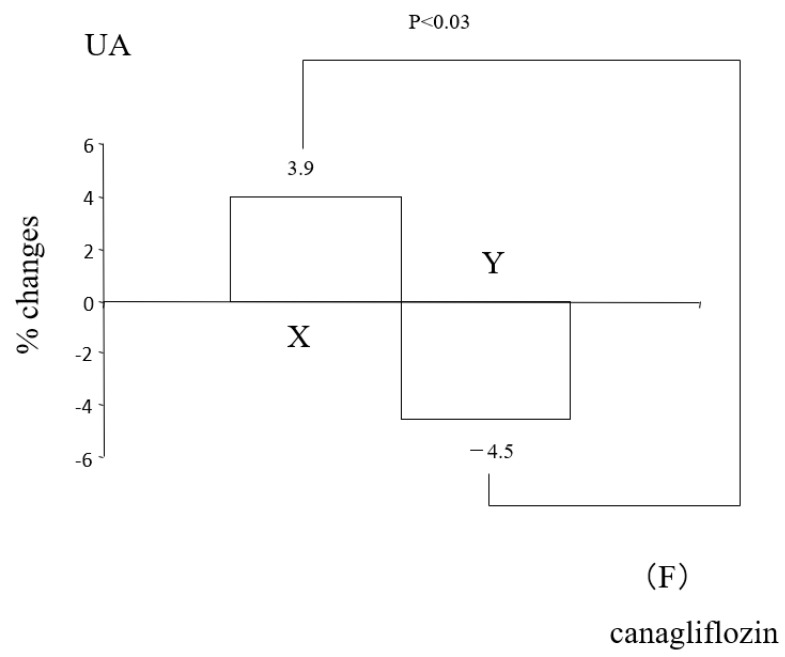
Differential effects on diabetic parameters by the changes in insulin resistance. ANCOVA was performed to analyze the inter-group differences in the changes in the indicated parameters in each treatment group (% changes). (**A**) HbA1c with tight Japanese diet; (**B**) HbA1c with pioglitazone; (**C**) UA with pioglitazone; (**D**) BMI with pioglitazone; (**E**) FBG with canagliflozin; (**F**) UA with canagliflozin.

Under the tight Japanese diet (X/Y = 33/32), comparable reductions in BMI were observed in both groups (Table 6(AX,AY)). In group X (Table 6(AX)), significant decreases were seen in HOMA-R, FBG, HbA1c, non-HDL-C, and insulin, while significant increases were observed in UA. T-C displayed a tendency to decrease. In group Y (Table 6(AY)), significant decreases were seen in HbA1c (not FBG), while significant increases were observed in HOMA-R, HOMA-B, and insulin. Significant inter-group differences were seen in the changes in HbA1c (greater reductions in group X versus Y, Figure 2A).

With pioglitazone (X/Y = 35/35), in group X (Table 6(BX)), significant decreases were observed in HOMA-R, FBG, HbA1c, insulin, TG, and TG/HDL-C, while significant increases were seen in HOMA-B, HDL-C, and BMI. Non-HDL-C exhibited a tendency to decrease. In group Y (Table 6(BY)), significant decreases were observed in HbA1c (not FBG), while significant increases were seen in HOMA-R, HOMA-B, HDL-C, BMI, and insulin. T-C displayed a tendency to increase in this group. Significant inter-group differences were observed in the changes in HbA1c and UA (greater reductions in group X versus group Y, Figure 2B,C) and BMI (greater increases in group X versus group Y, Figure 2D).

With canagliflozin (X/Y = 39/38), in group X (Table 6(CX)), significant decreases were observed in HOMA-R, FBG, HbA1c, insulin, TG, TG/HDL-C, and BMI. In group Y (Table 6(CY)), significant decreases were observed in FBG, HbA1c, and BMI, while significant increases were seen in HOMA-R, insulin, and HOMA-B. UA exhibited a tendency to decrease. Significant inter-group differences were observed in the changes in FBG (greater reductions in group X versus Y, Figure 2E) and UA (greater reductions in group Y versus X, Figure 2F). No inter-group differences were noted in the changes in HbA1c or BMI between these two groups (Table 6(CX,CY))

## 4. Discussion

### 4.1. Characteristics of Diabetic Parameters in Newly Diagnosed, Drug-Naïve Japanese Patients with T2DM

Notably, the glycemic control of newly diagnosed, untreated Japanese patients with T2DM is considerably poor, evidenced by an elevated HbA1c close to 10% (Table 1). This poor glycemic control can be attributed, in part, to the asymptomatic nature of this disorder, often described as a “silent killer,” wherein patients may not actively seek medical attention, resulting in delayed diagnosis and intervention.

HOMA-R exceeding 2.5 generally signifies the presence of insulin resistance [10,15], while HOMA-B below 30% indicates low beta-cell function [10,15]. Obesity is defined as BMI above 25 [8]. Considering these contextual factors, it is postulated that newly diagnosed, untreated Japanese patients with T2DM exhibit characteristics of high insulin resistance, relatively preserved beta-cell function, modest overweight status, and poor glycemic control. Thus, ameliorating insulin resistance is important in treating such populations. There are several strategies to reduce insulin resistance, either causally or consequentially, including a very low-calorie diet [11], pioglitazone [4,7], or SGLT-2 inhibitors [5].

Table 2 illustrates correlations between baseline levels of insulin resistance and various diabetic parameters in the overall subject cohort. These findings suggest a robust association between insulin resistance and FBG, insulin sensitivity, beta-cell function, body weight, and UA, with varying degrees of correlation with certain lipid profiles (TG, HDL-C, TG/HDL-C). The analysis in Table 3, stratifying subjects based on baseline insulin resistance levels, further supports these results. The dissociation of FBG and HbA1c concerning baseline insulin resistance may stem from the characteristic that insulin resistance may exert a lesser impact on postprandial glucose levels, while maintaining minimal glucose levels during fasting or other occasions is crucial in the early stages of diabetic history. It would be intriguing to compare data from newly diagnosed T2DM patients in other populations (e.g., Caucasians, Africans) to determine whether similar patterns emerge.

### 4.2. Link between Changes in Insulin Resistance and Diabetic Parameters

All three therapeutic strategies demonstrated a baseline-dependent regulation of insulin resistance, as illustrated in Figure 1A–C. After dividing subjects based on the median changes in HOMA-R values (ΔHOMA-R), group X (lower ΔHOMA-R) displayed a decrease, while group Y (higher ΔHOMA-R) exhibited an increase in HOMA-R (Table 6(AX,AY,BX,BY,CX,CY)). It is noteworthy that baseline HOMA-R was significantly higher in group X compared to group Y in each treatment group (results not presented in the table). These findings suggest that high baseline insulin resistance decreases while low baseline insulin resistance increases with each therapeutic approach. An intriguing observation is the substantial proportion of the population displaying an increase in insulin resistance. In this study, we investigated the changes in various diabetic parameters based on alterations in insulin resistance.

#### 4.2.1. FBG

Significant correlations between changes in insulin resistance (evaluated by HOMA-R) and changes in FBG were observed in all three treatment groups. Substantiating this, when subjects were divided into two groups based on changes in HOMA-R, reductions in FBG were observed only in those with decreased HOMA-R with a tight Japanese diet and pioglitazone (group X, Table 6(AX,AY,BX,BY)). With canagliflozin, significant reductions in FBG were observed in both groups (Table 6(CX,CY)), but greater reductions were seen in those with decreased insulin resistance (group X versus group Y, Figure 2E). Collectively, these findings strongly suggest a tight connection between insulin resistance and FBG. However, the causative relationship remains undetermined. To establish causation, it is imperative to consider the temporal sequence of events.

(1) With a tight Japanese diet, initial reductions in post-meal glucose (reduced input) occur, followed by the amelioration of glucotoxicity (reduction in insulin resistance and the enhancement of beta-cell function). In the long term, reduced caloric intake leads to weight loss, subsequently decreasing insulin resistance and FBG.
(2) With pioglitazone, an initial reduction in insulin resistance occurs, followed by decreases in blood glucose levels. In the long term, enhanced insulin sensitivity may lead to weight gain and ameliorate beta-cell dysfunction.
(3) With SGLT-2 inhibitors, as anticipated from their mode of action, initial reductions in both fasting and post-meal glucose (increased output) occur. Subsequently, glucotoxicity is alleviated (reduction in insulin resistance and the enhancement of beta-cell function). In the long term, weight reduction follows, leading to decreases in insulin resistance and blood glucose levels.

While it is widely accepted that reductions in insulin resistance cause decreases in blood glucose, it is still plausible that reductions in blood glucose cause decreases in insulin resistance, as described above. Therefore, observed correlations or effects do not necessarily imply causation. Conversely, the absence of correlations or effects does not definitively rule out a causal link, as confounding factors may mask these relationships. Further well-validated basic and clinical research is required to investigate this matter.

#### 4.2.2. HbA1c

Distinct outcomes were observed regarding HbA1c compared to FBG. While significant correlations were noted between changes in HOMA-R and HbA1c with a tight Japanese diet (R = 0.256, Table 5A) and pioglitazone (R = 0.266, Table 5B), no correlations were evident with canagliflozin (R = 0.118, Table 5C).

In a separate analysis, the SGLT-2 inhibitor canagliflozin exhibited distinct regulatory patterns compared to tight Japanese diet or pioglitazone. When subjects were stratified based on changes in insulin resistance, both tight Japanese diet and pioglitazone resulted in HbA1c reductions in both groups, with notable inter-group differences (higher reductions in HbA1c observed in those with greater reductions in insulin resistance, Table 6(AX,AY,BX,BY), Figure 2A,B). Conversely, with canagliflozin, similar, significant reductions in HbA1c were consistent regardless of changes in HOMA-R (Table 6(CX,CY)). The mechanism of action of SGLT2 inhibitors, independent of insulin secretion or action, implies that their efficacy remains unchanged irrespective of the status of insulin resistance and/or impaired beta-cell dysfunction. This could contribute to the lack of correlations between changes in HOMA-R and HbA1c with canagliflozin.

#### 4.2.3. Beta-Cell Function

Across all three therapeutic strategies, there were notable reductions in insulin resistance and increases in beta-cell function (assessed with HOMA-B). Supporting the notion that beta-cell function is stimulated in response to insulin resistance, significant correlations were observed between changes in insulin resistance (ΔHOMA-R) and beta-cell function (ΔHOMA-B, Table 5A–C). However, beta-cell function displayed distinct regulatory patterns based on changes in insulin resistance, as elucidated below: HOMA-B was significantly up-regulated in those with elevated insulin resistance in all three strategies (group Y, Table 6(AY,BY,CY)). Conversely, it exhibited different patterns in those with reduced insulin resistance (group X). With a tight Japanese diet, HOMA-B was significantly down-regulated (Table 6(AX)). By contrast, with pioglitazone, it was up-regulated (Table 6(BX)). With canagliflozin, there was a tendency to increase (Table 6(CX)). The mechanisms and implications of this divergent regulation in this subgroup are presently under investigation.

#### 4.2.4. Weight

Body weight management is crucial for obese patients with diabetes. It is well-established that excess weight exacerbates glucose control through deteriorated insulin resistance, and conversely, weight control positively impacts insulin sensitivity [16]. However, controversies surround this issue in pharmacotherapies. For instance, certain diabetes drugs like insulin or sulphonylurea have no impact on insulin resistance but induce weight gain [17]. DPP-4 inhibitors are considered neutral in weight or insulin sensitivity, but individuals responding efficiently to these drugs may experience weight gain [18,19]. SGLT-2 inhibitors reduce both weight and insulin resistance. However, previous findings have suggested that specific populations treated with SGLT-2 inhibitors may not experience weight loss, and correlations between changes in insulin resistance and weight are not consistently observed [8]. A TZD drug, such as pioglitazone, reduces insulin resistance but contributes to weight gain [4,6]. These complexities imply that weight loss (or gain) does not consistently correlate with decreased (or increased) insulin resistance during pharmacotherapies.

In this study, we explored the relationship between changes in insulin resistance and weight across three distinct therapeutic strategies, all of which aim to reduce insulin resistance.

(a) With a tight Japanese diet, similar weight reductions were observed irrespective of changes in insulin resistance (Table 6(AX,AY)). No correlations were identified between changes in insulin resistance and weight (Table 5A).
(b) With pioglitazone, on the contrary, more significant weight increases were noted in individuals with reduced insulin resistance (Figure 2D). Reductions in insulin resistance correlated with increased weight (Table 5B). The precise mechanism behind weight gain with pioglitazone remains unclear, but it has been hypothesized that the activation of PPARγ leads to an increase in the number and size of fat cells, resulting in increased fat storage [20]. In addition, the improvement in insulin sensitivity with this drug (referred to as group X in this paper) is accompanied by an increase in weight due to heightened lipogenesis [8]. These could contribute to an overall gain in body fat, leading to increased body weight.
(c) With canagliflozin, changes in insulin resistance were not associated with changes in weight (Table 5C). Irrespective of changes in insulin resistance with this drug, similar and significant reductions in weight were observed (Table 6(CX,CY)).

These findings challenge the conventional notion that increased weight worsens insulin resistance, while weight reduction improves it. There are several assumptions to explain these discrepancies. In human physiology, feedback mechanisms operate in many instances. In our results, body weight reduction with a tight Japanese diet and/or SGLT-2 inhibitor may activate feedback mechanisms that attempt to increase insulin resistance and conserve glucose.

#### 4.2.5. Lipids

Diabetic dyslipidemia is typically characterized by increased TG and reduced HDL-C [21]. Non-HDL-C is frequently increased and considered a better parameter for atherogenic lipid than LDL-C [21]. In this study, changes in insulin resistance with these three strategies resulted in differential correlations or regulations among the lipid parameters in relation to insulin resistance, as indicated below.

(a) With a tight Japanese diet, favorable effects on T-C or non-HDL-C were observed, as expected from the components of the Japanese diet (Table 4A). However, no correlations or changes in lipid parameters were noted, irrespective of changes in insulin resistance (Table 5A and Table 6(AX,AY)).
(b) With pioglitazone, the significant down-regulation of TG and up-regulation of HDL-C were observed (Table 4B), consistent with other reports [22]. Changes in insulin resistance correlated with changes in T-C, TG, and non-HDL-C (Table 5B) but not with HDL-C (Table 5B). Significant reductions in TG and TG/HDL-C were observed in individuals with reduced insulin resistance (group 6BX). Collectively, pioglitazone appears to have favorable effects on certain lipid parameters, and the reductions in these lipids seem to be linked to reductions in insulin resistance.
(c) Effects on lipids with SGLT-2 inhibitors are controversial [23]. In this study, with canagliflozin, the insignificant down-regulation of TG and significant up-regulation of HDL-C were observed (Table 4C). Changes in insulin resistance had no correlation with changes in HDL-C but showed a tendency to correlate with changes in TG (Table 5C). TG significantly decreased only in individuals with reduced insulin resistance (group 6CX). Thus, it appears that the modulation of insulin resistance with this SGLT-2 inhibitor is somewhat associated with TG but is not clearly associated with other lipid parameters.

#### 4.2.6. UA

In comparison to other diabetic parameters such as weight or lipids, UA is less well studied regarding its involvement in T2DM or insulin resistance. UA can impair insulin signaling pathways and interfere with insulin’s ability to regulate glucose metabolism [24,25]. Besides this, UA can promote inflammation, oxidative stress, and endothelial dysfunction, which contribute to the development of insulin resistance and beta-cell dysfunction [24,25]. However, the exact nature of this relationship is complex and not fully understood. In this study, the baseline UA had significant correlations with that of insulin resistance (Table 2). Another analysis showed that UA is more elevated in those with higher vs. lower baseline insulin resistance (Table 3). These results strongly argue that insulin resistance and UA are linked. However, it remains unclear whether insulin resistance causes the elevation of UA or the other way around. The therapeutic strategies in this present study all reduced insulin resistance; however, distinct UA regulatory patterns were seen as described below. (1) Weight reductions resulted in reduced insulin resistance and UA [26]. However, with a tight Japanese diet, unexpectedly and surprisingly, UA was significantly increased though reductions in body weight and insulin resistance (Table 4A). Further, reductions in insulin resistance appear to be negatively correlated to UA (Table 5A). Those with reduced insulin resistance had increased UA, though these subjects had reduced weight (Table 6AX). This may be due to the fact that the Japanese diet contains high UA [11,12]. It is of interest to evaluate this using other diets (e.g., Mediterranean). (2) With pioglitazone or canagliflozin, changes in insulin resistance may not have significant correlations or effects on UA (Table 5B,C and Table 6(BX,BY,CX,CY)). However, UA regulatory patterns depending on the changes in insulin resistance are distinct between these two drugs; relative reductions or increases in UA were observed in those with reduced insulin resistance in pioglitazone or canagliflozin, respectively (Figure 2C,F). Some diabetes drugs including DPP-4 inhibitors are known to elevate UA [13]. It is possible that reduced blood glucose levels per se somehow increase UA through reduced excretion or increased re-absorption in the kidneys. Taken together, these results indicate that UA regulation is rather complex, and in addition to insulin resistance, other mechanisms may be involved in the regulation of UA during therapies in T2DM. Basic research is required to investigate this issue.

### 4.3. Limitations of This Study

Certain drawbacks or limitations exist in this study. It is an observational study with a relatively small number of subjects and short study duration. Additionally, there is a gender disparity in the number of subjects and dosing of the drugs. The insulin resistance-lowering mechanisms of the therapies in this study (low-calorie diet, pioglitazone, or canagliflozin) are distinct. Therefore, it may not be appropriate to directly compare them. It remains to be investigated whether similar or different results would be observed in other populations. Thus, the results presented in this study might only be considered “hypothesis-generating”. To prove the credibility of these results, randomized controlled trials in different diabetic populations are required. However, this could be expensive, time consuming, and, on some occasions, unethical. In observational studies, randomization may naturally occur. Further, based on the design of the protocol (monotherapy in drug-naïve subjects), the observed results were most probably caused exclusively by the treatments undertaken (tight Japanese diet, pioglitazone, or canagliflozin).

## 5. Conclusions

In conclusion, the investigation into the impacts of a very low-calorie (tight) Japanese diet, the thiazolidinedione (TZD) pioglitazone, and the sodium-glucose cotransporter-2 (SGLT-2) inhibitor canagliflozin unveiled a collective reduction in insulin resistance. The significant negative correlations observed between the changes in insulin resistance, as assessed by HOMA-R, and the baseline insulin resistance indicate a tendency for individuals with high insulin resistance to experience a decrease, while those with low insulin resistance may exhibit an increase (regression to the means). Unexpectedly, a noteworthy proportion of subjects demonstrated an increase in insulin resistance with these therapeutic strategies. The analyses conducted in this study revealed that, while significant correlations were identified between the changes in insulin resistance and FBG, insulin levels, or beta-cell function, other parameters such as HbA1c, body weight, some lipids, or uric acid (UA) displayed distinct regulatory patterns contingent upon the type of therapy employed. Stratifying subjects into two groups based on the median value of the changes in HOMA-R in each group-lower half (group X) and upper half (group Y)-revealed divergent regulations in FBG, beta-cell function, certain lipids, body weight, and UA.

## Figures and Tables

**Table 1:** The baseline characteristics of all subjects encompassed in this study (*n* = 212).

	Baseline
F/M	49/163
age	52.3 ± 12.6
FBG (mg/dL)	202.6 ± 56.3
HbA1c (%)	9.75 ± 1.96
insulin (μL/mL)	7.44 ± 5.05
HOMA-R	3.64 ± 2.52
HOMA-B	23.23 ± 20.44
BMI	25.89 ± 4.93
T-C (mg/dL)	215.3 ± 42.2
TG (mg/dL)	181.7 ± 160.4
HDL-C (mg/dL)	52.6 ± 12.8
non-HDL-C (mg/dL)	148.0 ± 61.8
TG/HDL-C	3.86 ± 4.16
UA (mg/dL)	4.96 ± 1.33

**Table 2:** Correlations between insulin resistance (HOMA-R) and diabetic parameters at baseline (all the subjects).

Baseline HOMA-R vs. Baseline	R	*p*-Values
age	−0.145	<0.04
FBG (mg/dL)	0.295	<0.00001
HbA1c (%)	0.082	n.s.
insulin (μL/mL)	0.886	<0.00001
HOMA-B	0.535	<0.00001
BMI	0.466	<0.00001
T-C (mg/dL)	−0.019	n.s.
TG (mg/dL)	0.127	0.064
HDL-C (mg/dL)	−0.131	0.056
nonHDL-C (mg/dL)	0.019	n.s.
TG/HDL-C	0.12	0.0817
UA (mg/dL)	0.279	0.00001

Simple regression analysis was performed between HOMA-R and indicated diabetic parameters at baseline.

**Table 3:** Comparison of baseline diabetic parameters depending on insulin resistance (all the subjects).

	A	B	*p*-Values
N	107	105	n.s.
age	54.1 ± 11.4	50.6 ± 13.5	<0.05
FBG (mg/dL)	191.8 ± 54.4	213.6 ± 56.4	<0.005
HbA1c (%)	9.72 ± 2.10	9.78 ± 1.82	n.s.
insulin (μL/mL)	4.13 ± 2.35	10.81 ± 4.83	<0.00001
HOMA-R	1.87 ± 1.04	5.46 ± 2.29	<0.00001
HOMA-B	14.68 ± 11.89	31.94 ± 23.48	<0.00001
BMI	24.03 ± 4.03	27.79 ± 5.07	<0.00001
T-C (mg/dL)	219.4 ± 45.5	211.2 ± 38.3	n.s.
TG (mg/dL)	163.7 ± 176.0	200.0 ± 141.3	0.099
HDL-C (mg/dL)	54.5 ± 14.4	50.7 ± 10.7	<0.04
nonHDL-C (mg/dL)	137.8 ± 74.7	160.5 ± 37.8	0.051
TG/HDL-C	3.32 ± 4.16	4.40 ± 4.09	0.058
UA (mg/dL)	4.69 ± 1.25	5.25 ± 1.36	<0.003

Unpaired Student’s *t*-test was used to compare the baseline characteristics of the indicated diabetic parameters depending on the degree of baseline insulin resistance. The subjects were divided into two groups according to the median values of the baseline HOMA-R (lower half: group A and upper half: group B).

**Table 4:** Changes in diabetic parameters with tight Japanese diet, pioglitazone, or canagliflozin. (Panel **A**) tight Japanese diet; (Panel **B**) pioglitazone; (Panel **C**) canagliflozin.

**(A)**				
	**Baseline**	**3 Months**	***p*-Values**	**% Changes**
F/M	15/50			
age	50.8 ± 12.9			
FBG (mg/dL)	189.4 ± 49.3	167.6 ± 51.9	<0.0004	−11.5
HbA1c (%)	9.08 ± 1.32	7.96 ± 1.52	<0.00001	−12.3
insulin (μL/mL)	8.04 ± 5.25	7.20 ± 5.02	n.s.	−10.4
HOMA-R	3.80 ± 2.28	2.95 ± 2.18	<0.01	−22.3
HOMA-B	26.23 ± 19.94	30.60 ± 24.92	<0.05	16.6
BMI	26.20 ± 4.97	25.29 ± 4.77	<0.00001	−3.4
T-C (mg/dL)	208.0 ± 31.7	201.6 ± 33.7	*p* < 0.05	−3
TG (mg/dL)	157.2 ± 100.9	142.2 ± 85.6	n.s.	−9.5
HDL-C (mg/dL)	53.2 ± 11.7	53.2 ± 11.5	n.s.	0
nonHDL-C (mg/dL)	116.9 ± 71.7	110.8 ± 70.2	<0.03	−5.2
TG/HDL-C	3.18 ± 2.28	2.87 ± 2.05	n.s.	−9.7
UA (mg/dL)	4.84 ± 1.39	5.13 ± 1.47	<0.002	5.9
**(** **B** **)**				
	**Baseline**	**3 Months**	***p*-Values**	**% Changes**
F/M	17/53			
age	53.0 ± 11.7			
HOMA-R	3.62 ± 2.27	2.64 ± 1.78	<0.00001	−27
FBG (mg/dL)	214.4 ± 53.1	170.2 ± 63.3	<0.00001	−20.6
HbA1c (%)	9.85 ± 1.60	8.37 ± 1.69	<0.00001	−15
insulin (μL/mL)	6.93 ± 4.36	6.61 ± 4.70	n.s.	−4.6
HOMA-B	19.00 ± 15.42	32.31 ± 38.79	<0.003	70
BMI	25.20 ± 5.23	25.64 ± 5.30	<0.00001	1.7
T-C (mg/dL)	210.3 ± 37.8	213.0 ± 36.2	n.s.	1.2
TG (mg/dL)	177.9 ± 122.6	145.4 ± 88.7	<0.0007	−18.2
HDL-C (mg/dL)	49.9 ± 11.3	56.5 ± 16.0	<0.00001	13.2
nonHDL-C (mg/dL)	160.4 ± 38.7	156.44 ± 38.8	n.s.	−2.4
TG/HDL-C	3.56 ± 10.7	2.57 ± 5.52	<0.0002	−27.8
UA (mg/dL)	4.67 ± 1.31	4.64 ± 1.21	n.s.	−0.6
**(C)**				
	**Baseline**	**3 Months**	***p*-Values**	**% Changes**
F/M	18/59			
age	53.5 ± 12.5			
HOMA-R	3.53 ± 2.72	2.48 ± 2.05	<0.00001	−29.7
FBG (mg/dL)	203.1 ± 64.8	150.6 ± 47.9	<0.00001	−25.8
HbA1c (%)	10.24 ± 2.61	8.34 ± 1.97	<0.00001	−18.5
insulin (μL/mL)	7.38 ± 5.92	6.85 ± 6.28	n.s.	−7.1
HOMA-B	24.53 ± 26.76	33.46 ± 43.00	<0.00001	36.4
BMI	26.26 ± 5.64	25.82 ± 5.66	<0.00001	−1.6
T-C (mg/dL)	226.1 ± 49.7	224.2 ± 46.1	n.s.	−0.8
TG (mg/dL)	205.3 ± 213.0	193.1 ± 233.7	0.087	−5.9
HDL-C (mg/dL)	54.0 ± 14.4	56.2 ± 14.8	<0.03	4
nonHDL-C (mg/dL)	167.7 ± 58.7	163.7 ± 54.7	n.s.	−2.3
TG/HDL-C	4.44 ± 5.77	4.19 ± 7.24	n.s.	−5.6
UA (mg/dL)	5.33 ± 1.25	5.31 ± 1.26	n.s.	−0.3

Paired Student’s *t*-test was used to compare the changes in the indicated parameters after 3 months of treatment with very low-calorie (tight) Japanese diet, pioglitazone, or canagliflozin. The results are expressed as the mean + SD.

**Table 5:** Correlation of the changes in insulin resistance and those of other diabetic parameters. (**A**) Tight Japanese diet; (**B**) pioglitazone; (**C**) canagliflozin.

**(** **A)**		
**Δ** **HOMA-R vs.**	**R**	***p*** **-Values**
baseline HOMA-R	−0.688	<0.00001
ΔFBG	0.599	<0.00001
ΔHbA1c	0.256	<0.04
Δinsulin	0.932	<0.00001
ΔHOMA-B	0.452	<0.0002
ΔBMI	0.102	n.s.
ΔT-C	−0.078	n.s.
ΔTG	0.137	n.s.
ΔHDL-C	0.022	n.s.
ΔnonHDL-C	−0.091	n.s.
ΔTG/HDL-C	0.154	n.s.
ΔUA	−0.217	0.082
**(B)**		
**Δ** **HOMA-R vs.**	**R**	***p*** **-Values**
baseline HOMA-R	−0.654	<0.00001
ΔFBG	0.51	<0.00001
ΔHbA1c	0.266	<0.03
Δinsulin	0.771	<0.00001
ΔHOMA-B	0.298	<0.02
ΔBMI	−0.342	<0.04
ΔT-C	0.283	<0.02
ΔTG	0.299	<0.02
ΔHDL-C	0.087	n.s.
ΔnonHDL-C	0.26	<0.03
ΔTG/HDL-C	0.23	0.055
ΔUA	0.077	n.s.
**(** **C)**		
**Δ** **HOMA-R vs.**	**R**	***p*** **-Values**
baseline HOMA-R	−0.685	<0.00001
ΔFBG	0.322	<0.007
ΔHbA1c	0.118	n.s.
Δinsulin	0.849	<0.00001
ΔHOMA-B	0.365	<0.004
ΔBMI	−0.178	n.s.
ΔT-C	0.041	n.s.
ΔTG	0.215	0.072
ΔHDL-C	−0.041	n.s.
ΔnonHDL-C	0.06	n.s.
ΔTG/HDL-C	0.293	<0.02
ΔUA	−0.196	0.093

Simple regression analysis was performed between the changes in (Δ) HOMA-R and those of diabetic parameters.

**Table 6:** Effect of tight Japanese diet, pioglitazone, or canagliflozin on diabetic parameters in two groups with distinct changes in insulin resistance. (**A**) Tight Japanese diet (group X and Y); (**B**) pioglitazone (group X and Y); (**C**) canagliflozin (group X and Y).

**(AX)**				
	**Baseline**	**3 Months**	***p*-Values**	**% Changes**
N	33			
age	51.0 ± 13.4			
HOMA-R	5.47 ± 2.89	2.83 ± 1.86	<0.00001	−48.2
FBG (mg/dL)	205.6 ± 47.8	155.9 ± 39.4	<0.00001	−24.1
HbA1c (%)	9.31 ± 1.37	7.79 ± 1.43	<0.00001	−16.3
insulin (μL/mL)	10.92 ± 5.27	7.23 ± 4.00	<0.00001	−33.7
HOMA-B	31.70 ± 21.67	20.59 ± 16.48	<0.00001	−35
BMI	27.65 ± 4.79	26.59 ± 4.88	<0.00001	−3.8
T-C (mg/dL)	208.4 ± 31.4	200.7 ± 37.5	0.094	−3.6
TG (mg/dL)	171.0 ± 81.5	153.4 ± 81.3	n.s.	−10.2
HDL-C (mg/dL)	52.0 ± 9.4	52.7 ± 11.4	n.s.	1.3
nonHDL-C (mg/dL)	156.3 ± 29.1	148.0 ± 33.8	<0.05	−5.3
TG/HDL-C	3.49 ± 1.95	3.08 ± 1.90	n.s.	−11.7
UA (mg/dL)	4.93 ± 1.45	5.40 ± 1.59	<0.0002	9.5
**(AY)**				
	**Baseline**	**3 Months**	***p*-Values**	**% Changes**
N	32			
age	50.6 ± 12.7			
HOMA-R	2.08 ± 1.35	3.08 ± 2.50	<0.01	48
FBG (mg/dL)	172.6 ± 45.6	179.7 ± 60.4	n.s.	4.1
HbA1c (%)	8.85 ± 1.25	8.15 ± 1.60	<0.00001	−7.9
insulin (μL/mL)	5.07 ± 3.22	7.16 ± 5.96	<0.02	41.2
HOMA-B	20.59 ± 16.48	28.39 ± 26.80	<0.04	37.8
BMI	24.71 ± 4.77	23.96 ± 4.33	<0.007	−3
T-C (mg/dL)	207.6 ± 32.6	202.6 ± 29.9	n.s.	−2.4
TG (mg/dL)	143.0 ± 117.3	130.6 ± 89.6	n.s.	−8.6
HDL-C (mg/dL)	54.4 ± 13.7	53.8 ± 11.7	n.s.	−1.1
nonHDL-C (mg/dL)	153.1 ± 31.1	148.8 ± 28.8	n.s.	−2.8
TG/HDL-C	2.85 ± 2.58	2.66 ± 2.21	n.s.	−6.6
UA (mg/dL)	4.75 ± 1.35	4.86 ± 1.31	n.s.	2.3
**(** **BX)**				
	**Baseline**	**3 Months**	***p*-Values**	**% Changes**
N	35			
age	52.2 ± 11.9			
HOMA-R	5.03 ± 2.15	2.52 ± 1.58	<0.00001	−49.9
FBG (mg/dL)	223.1 ± 58.1	150.7 ± 50.0	<0.00001	−32.4
HbA1c (%)	9.78 ± 1.59	7.93 ± 1.68	<0.00001	−18.9
insulin (μL/mL)	9.55 ± 4.39	6.88 ± 3.70	<0.00001	−27.9
HOMA-B	26.07 ± 18.21	35.02 ± 23.19	<0.007	34.3
BMI	26.21 ± 5.44	26.77 ± 5.58	<0.00001	2.1
T-C (mg/dL)	216.0 ± 42.7	211.0 ± 40.7	n.s.	−2.3
TG (mg/dL)	207.8 ± 147.4	151.4 ± 101.4	<0.0004	−27.1
HDL-C (mg/dL)	48.7 ± 10.5	54.2 ± 13.4	<0.0005	11.2
nonHDL-C (mg/dL)	167.2 ± 40.6	156.8 ± 40.9	0.052	−6.2
TG/HDL-C	4.73 ± 4.01	3.10 ± 2.53	<0.001	−34.4
UA (mg/dL)	5.12 ± 1.42	4.98 ± 1.20	n.s.	−2.7
**(BY)**				
	**Baseline**	**3 Months**	***p*-Values**	**% Changes**
N	35			
age	53.8 ± 11.6			
HOMA-R	2.22 ± 1.33	2.76 ± 1.97	<0.02	24.3
FBG (mg/dL)	205.7 ± 46.8	189.6 ± 69.6	0.082	−7.8
HbA1c (%)	9.91 ± 1.64	8.81 ± 1.61	<0.00001	−11
insulin (μL/mL)	4.32 ± 2.30	6.35 ± 5.56	<0.01	46.9
HOMA-B	11.93 ± 6.92	29.59 ± 50.01	<0.04	148
BMI	23.89 ± 4.09	24.25 ± 4.19	<0.00001	1.5
T-C (mg/dL)	204.7 ± 31.7	215.0 ± 31.7	0.078	5
TG (mg/dL)	144.3 ± 81.1	133.5 ± 70.9	n.s.	−7.4
HDL-C (mg/dL)	51.7 ± 12.2	59.2 ± 18.2	<0.0005	14.5
nonHDL-C (mg/dL)	153.6 ± 35.9	156.0 ± 37.2	n.s.	1.5
TG/HDL-C	2.93 ± 1.83	2.60 ± 1.76	n.s.	−11.2
UA (mg/dL)	4.22 ± 1.03	4.30 ± 1.13	n.s.	1.8
**(** **CX)**				
	**Baseline**	**3 Months**	***p*-Values**	**% Changes**
N	39			
age	52.46 ± 13.05			
HOMA-R	4.79 ± 2.74	2.32 ± 1.75	<0.00001	−51.5
FBG (mg/dL)	220.0 ± 61.6	143.6 ± 31.5	<0.00001	−34.7
HbA1c (%)	10.75 ± 2.74	8.59 ± 2.18	<0.00001	−20
insulin (μL/mL)	9.40 ± 6.23	6.64 ± 5.26	<0.00001	−29.3
HOMA-B	27.95 ± 28.09	33.30 ± 28.94	n.s.	19.1
BMI	26.88 ± 4.67	26.50 ± 5.01	<0.05	−1.4
T-C (mg/dL)	223.7 ± 48.4	220.0 ± 44.7	n.s.	−1.6
TG (mg/dL)	201.5 ± 133.5	175.0 ± 123.0	<0.04	−13.1
HDL-C (mg/dL)	53.0 ± 15.7	55.1 ± 15.8	n.s.	3.9
nonHDL-C (mg/dL)	170.7 ± 50.7	165.0 ± 46.0	n.s.	−3.3
TG/HDL-C	4.47 ± 4.73	3.70 ± 3.84	<0.02	−17.2
UA (mg/dL)	5.13 ± 1.14	5.33 ± 1.17	n.s.	3.8
**(C** **Y)**				
	**Baseline**	**3 Months**	***p*-Values**	**% Changes**
N	38			
age	53.71 ± 13.32			
HOMA-R	2.25 ± 1.30	2.65 ± 1.97	<0.05	17.7
FBG (mg/dL)	185.7 ± 59.7	157.9 ± 57.5	<0.0002	−14.9
HbA1c (%)	9.71 ± 2.14	8.09 ± 1.76	<0.0001	−16.6
insulin (μL/mL)	5.31 ± 3.52	7.07 ± 5.37	<0.01	33.1
HOMA-B	21.02 ± 19.03	33.63 ± 32.56	<0.007	59.9
BMI	25.61 ± 4.53	25.13 ± 4.27	<0.0004	−1.8
T-C (mg/dL)	228.4 ± 54.4	228.6 ± 49.6	n.s.	0
TG (mg/dL)	213.5 ± 283.5	211.6 ± 319.7	n.s.	−0.8
HDL-C (mg/dL)	55.5 ± 13.4	57.3 ± 13.8	n.s.	3.2
nonHDL-C (mg/dL)	172.8 ± 55.4	171.2 ± 51.2	n.s.	−0.9
TG/HDL-C	4.45 ± 6.72	4.69 ± 9.60	n.s.	5.3
UA (mg/dL)	5.54 ± 1.30	5.29 ± 1.37	0.079	−4.5

Paired Student’s *t*-test was used to compare the changes in the indicated parameters in two groups with distinct changes in insulin resistance. In each treatment group, the subjects were divided into two groups based on the median value of the changes (Δ) in HOMA-R (lower half: group X and upper half: group Y). The results are expressed as the mean + SD.

## Data Availability

The data that support the findings of this study are available from the corresponding author (E.K.) upon reasonable request.

## Abbreviations

T2DM	type 2 diabetes
SGLT-2	sodium-glucose co-transporter
BMI	body mass index
FBG	fasting blood glucose
HOMA-R	homeostasis model assessment-R
HOMA-B	homeostasis model assessment-B
T-C	total cholesterol
TG	triglyceride
HDL-C	high density lipoprotein cholesterol
UA	uric acid
